# Novel Approach to Sample Preconcentration by Solvent Evaporation in Flow Analysis

**DOI:** 10.3390/molecules25081886

**Published:** 2020-04-18

**Authors:** Justyna Paluch, Joanna Kozak, Marcin Wieczorek, Michał Woźniakiewicz, Małgorzata Gołąb, Ewelina Półtorak, Sławomir Kalinowski, Paweł Kościelniak

**Affiliations:** 1 Faculty of Chemistry, Jagiellonian University, Gronostajowa 2, 30-387 Krakow, Poland; justyna.paluch@uj.edu.pl (J.P.); marcin.wieczorek@uj.edu.pl (M.W.); michal.wozniakiewicz@uj.edu.pl (M.W.); mgolab@doctoral.uj.edu.pl (M.G.); ewelina.poltorak@o2.pl (E.P.); pawel.koscielniak@uj.edu.pl (P.K.); 2 Department of Chemistry, University of Warmia and Mazury, Plac Łódzki 4, 10-957 Olsztyn, Poland; kalinow@uwm.edu.pl

**Keywords:** preconcentration, evaporation, flow analysis, sequential injection analysis

## Abstract

A preconcentration module operated in flow mode and integrated with a sequential injection system with spectrophotometric detection was developed. Using the system, preconcentration was performed in continuous mode and was based on a membraneless evaporation process under diminished pressure. The parameters of the proposed system were optimized and the system was tested on the example of the spectrophotometric determination of Cr(III). The preconcentration effectiveness was determined using the signal enhancement factor. In the optimized conditions for Cr(III), it was possible to obtain the signal enhancement factors of around 10 (SD: 0.9, *n* = 4) and determine Cr(III) with precision and intermediate precision of 8.4 and 5.1% (CV), respectively. Depending on the initial sample volume, signal enhancement factor values of about 20 were achieved. Applicability of the developed preconcentration system was verified in combination with the capillary electrophoresis method with spectrophotometric detection on the example of determination of Zn in certified reference materials of drinking water and wastewater. Taking into account the enhancement factor of 10, a detection limit of 0.025 mg L^−1^ was obtained for Zn determination. Zn was determined with precision less than 6% (CV) and the results were consistent with the certified values.

## 1. Introduction

Sample pretreatment is an integral and indispensable stage of many analytical methods. Performed in a traditional mode, it is often laborious, multi-stage, and time-consuming, especially when analyte preconcentration is necessary before the determination. On the other hand, it is necessary to achieve the concentration of the analyte within the limits of quantification of the method, and the way of its implementation is important because of the need to obtain satisfactory precision and accuracy of analytical results. 

In recent years, special attention has been paid to the development of environmentally friendly analytical methods. The rules of green analytical chemistry (GAC) emphasize, among others, the importance of developing direct methods, integrating analytical operations, developing automated and/or miniaturized systems, using multi-analyte methods, reducing the use of toxic reagents and waste production, and ensuring work safety [1]. These principles should also be met at the stage of sample pretreatment [2]. Therefore, improving existing and developing new sustainable analytical procedures is becoming a significant trend in analytical chemistry [1,2,3,4,5].

In this field, the significance of flow techniques should be emphasized, which by enabling the automation (also miniaturization) of analytical procedures, integration of analytical operations, limiting the use of reagents, acceleration of analyses or improving work safety, meet the important principles of green analytical chemistry. The achievements in sample pretreatment performed using flow-based methods were presented in a large numbers of research articles, review papers [6,7,8,9,10,11,12,13], as well as in monographs [14,15,16,17,18,19]. 

The sample preparation step is considered as the crucial one in the light of GAC principles [2]. One of the ways used very often for sample preparation is sample preconcentration coupled with the analyte isolation from the sample matrix. It is usually performed by well-known extraction techniques (liquid–liquid, solid-phase) in various macro- and micro systems. In the literature one can find many examples of modern extraction approaches used for sample preparation [3,4], a number of them have been successfully adapted to flow analysis [6,7,8,9,10,13]. However, independently of the specificity of micro-extraction techniques, the necessity of using reagents, including organic solvents can be regarded as a drawback from the green chemistry point of view. 

One of few non-reagent-based sample preparation ways is the evaporation technique. The approach based on evaporation has been quite often implemented in flow systems equipped with pervaporation modules [20,21,22,23,24,25,26]. In the developed systems, temperature assisted sample preconcentration, analyte (analytes) separation, or analyte postconcentration was coupled to process of diffusion using properly selected permeable membranes. In these systems, it is important to select a membrane with the right properties to ensure good contact between the solution and the membrane, and adequate pervaporation efficiency. Various approaches to the sample preconcentration stage based on the pervaporation/evaporation have been also developed using microfluidic and paper-based devices [27,28,29]. Many of these approaches are characterized by high preconcentration factors. They are usually designed for specific determinations and cannot be used directly for other analytical determinations. 

Although the membraneless evaporation approach is still quite popular in batch analysis, only few proposals of flow-based membraneless systems have been reported in the literature. One of the reasons may be difficulties in designing appropriate modules ensuring obtaining controlled preconcentration factor values and sufficiently repeatable, and accurate analytical results. Membraneless evaporation systems have been developed for both preconcentrating the analyte [30] and separating the analyte from the sample matrix [31,32,33]. Regarding the flow injection (FI) system employing a membraneless evaporation for an analyte preconcentration, a method for the determination of anthocyanins in wine based on continuous liquid–solid extraction, evaporation, HPLC separation and photometric detection was developed [30]. In the system, the analytes were removed from the wine in a continuous way using a C_18_ minicolumn and the eluted fraction was preconcentrated by solvent evaporation assisted by heat and removing off the vapor using a flow of N_2_. Using the developed evaporation system, the extract was subjected to evaporation (5 min) at a temperature of 140 °C, with N_2_ flow equal to 600 mL min^−1^ and transported to the chromatographic system. Values of the coefficient of variance (CV) of the results of determination of anthocyanins were between 10% and 15% and the values of enrichment factor were not provided in the article. The second group of methods based on the membraneless evaporation, was developed to separate the (volatile) analyte from the sample matrix. To this aim, FI systems for the determination of ethanol in alcoholic drinks based on the reduction of dichromate by ethanol vapor [31,32] were developed. The system [31] was equipped with a module containing two parallel channels of appropriate configuration, applied as a donor and acceptor channel. Using the system, the detection limit about three times lower than using conventional gas diffusion unit was achieved. In [32], the diffusion of the volatile analyte took place in the space between specially designed two chambers. The chambers were additionally aerated to increase the diffusion. Detection limit was found to be 2.7% (*v*/*v*) of ethanol for diffusion time of 60 s and a precision of 3.7% CV (coefficient of variance). The approach based on the use of the evaporation unit described in [31] was also adapted for direct quantitation of calcium carbonate in cement with contactless conductivity detection [33]. The results of the determinations were obtained with the precision (CV) better than 5.3%. 

In the present paper, an instrumental system with an original preconcentration module operated in a flow mode and integrated with a sequential injection (SI) system with spectrophotometric detection has been proposed. The novelty of the proposed system consists in the possibility of preconcentrating the sample based on a membraneless evaporation under diminished pressure in a continuous mode, using a mechanized flow system. The system has the potential to be used for various samples and to perform the preconcentration from different initial sample volumes. Parameters of the operation of the evaporation module and the flow system were optimized. The operation of the developed system was tested on the example of preconcentration and determination of Cr(III) and verified by the determination of Zn in certified reference materials of drinking water and wastewater with the use of the capillary electrophoresis method. 

## 2. Results and Discussion 

Research included: Design of a flow system containing a module for preconcentration, optimization of evaporation conditions in order to achieve effective preconcentration factor with acceptable precision, testing, and verification of the system on the example of the determination of selected analytes. 

### 2.1. Evaporation Module 

**Figure 1:**
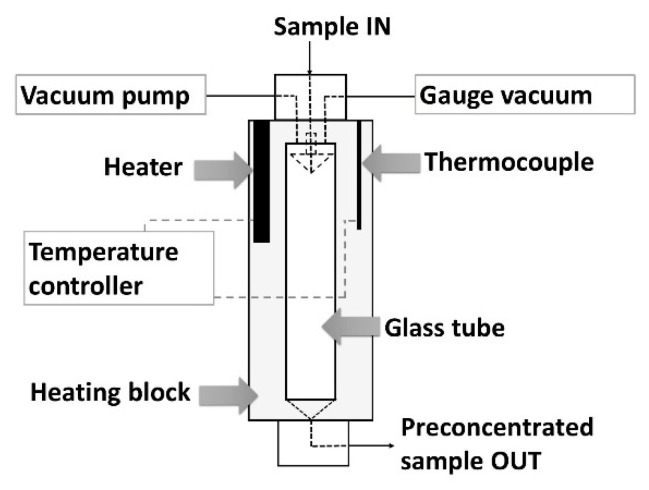
Scheme of the developed evaporation module.

**Figure A1:**
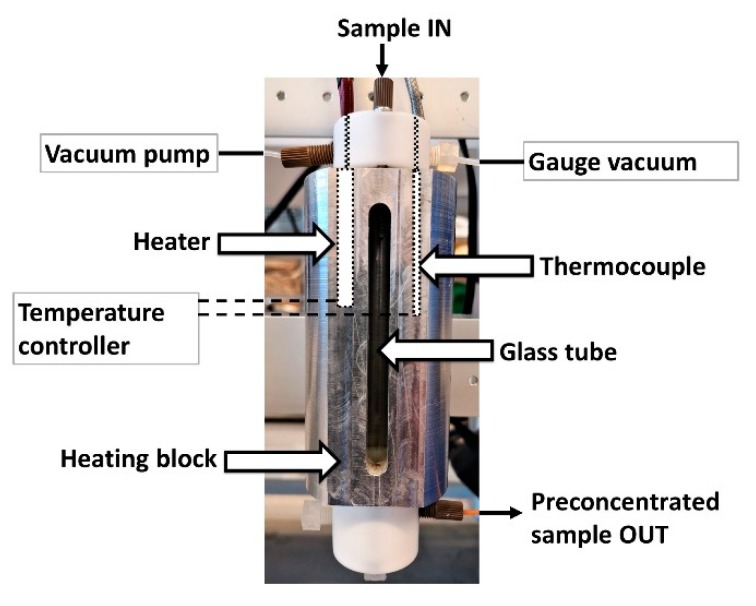
Evaporation module.

The main element of the constructed flow system was the module for preconcentration of a sample by evaporation under diminished pressure that is schematically shown in Figure 1 (and presented in Figure A1). The evaporation module (EM) consists of a glass tube with a capacity of about 15 mL (1.4 (i.d.) × 10 (height) cm), which is located inside an aluminum block. The block is responsible for thermostating the system in the temperature range from ambient to 150 °C. At the top the cylinder is covered with a PTFE block with an inlet (i.d. 0.8 mm) for dispensing solution of a sample. In the upper part there are also connections with a vacuum pump and a pressure gauge. The sample is introduced to the vessel through an inverted cone-shaped drop-forming element. Due to the selected height of the glass tube and the specific shape of this element the sample loss caused by its suction into the vacuum pump has been reduced. The lower part of the cylinder is closed by another PTFE block. The inner part of the bottom block is of the shape of an inverted cone with a narrow outlet (i.d. 0.8 mm) allowing even a small amount of preconcentrated sample to be collected and introduced into the flow system. During the evaporation process, the dosed drops of solution fell freely to the bottom of the vessel. Evaporation conditions (flow, pressure, and temperature) were selected so that the concentrated solution accumulated at the bottom of the vessel without filling it, so that as little solution as possible remained or splashed on the walls of the vessel.

### 2.2. Flow System 

**Figure 2:**
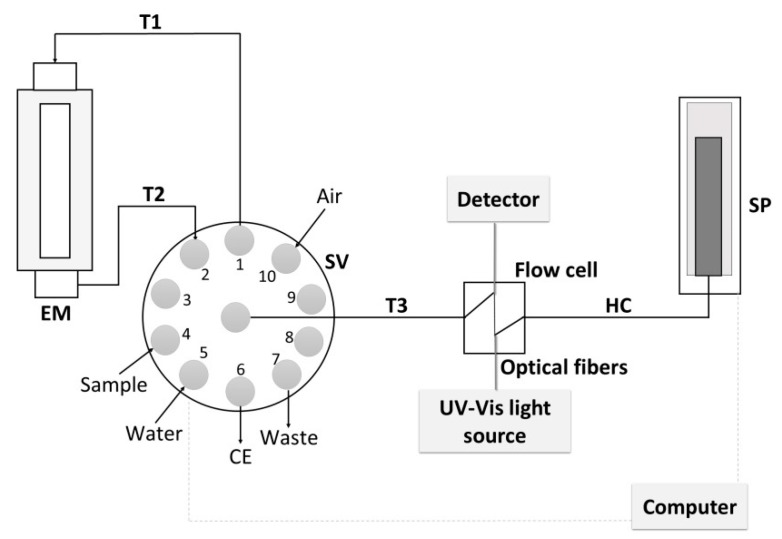
Scheme of the developed flow system with the evaporation module (EM); SV—selection valve, SP—syringe pump, HC—holding coil, T (1–3)—tubing, CE—capillary electrophoresis system.

A sequential injection analysis system was used to mechanize the sample evaporation process in the developed EM and to enable repeatable sampling from the module for measurement after the evaporation process. The scheme of the system is presented in Figure 2. The system was equipped with a pressure syringe pump (SP, with a capacity of syringe of 4 mL) enabling the collection of precisely defined portions of liquid from the EM to a flow cell with the selected flow rate. The use of a ten-position selection valve (SV) allowed control of the direction of the sample flow between individual elements of the system. The SV was connected to the upper part of the EM (Figure 2; tubing T1, 3 cm) and simultaneously with the lower part of the EM (tubing T2, 3 cm), and to the SP with PTFE tubing (i.d. 0.5 mm). A flow cell (1 cm wide) has been installed in the path between the SV (tubing T3, 3 cm) and the pump (holding coil, HC, 5 cm). 

### 2.3. Preconcentration Procedure 

The measurement procedure developed for the flow system with the EM for the evaporation of a 6 mL of sample is presented in Table 1. At the beginning, T3, HC, and SP were washed with the sample (Table 1, Steps 1,2). Next the defined volume of sample was introduced into the SP, and after reversing the flow direction, sample was dosed to EM (through the HC, T3, and T1) with a selected flow rate (Steps 3–6). The evaporation process was carried out under diminished pressure and at a selected temperature. Evaporation conditions (sample dosing flow rate, pressure and temperature) were selected so that the evaporation process took place during the introduction of the sample into the vessel. 

After completing the sample dispensing, the vacuum pump was turned off (the system was opened to equalize the pressure, Step 7, 30 s). Then a segment of air (1000 μL) was introduced into the tube T3 and HC (to prevent the dilution of the preconcentrated sample, Table 1, Step 8) and the sample was taken from the bottom of the vessel (through T2 and T3 tubing) to the flow cell and HC using the SP (Step 9)—to measure the signal for the sample after the preconcentration procedure. The segment of air was used as the carrier, its volume was selected so that after introducing the preconcentrated sample to the HC (after the evaporation process), air was moved to the SP and later (after reversing the flow direction) it allowed the whole sample to be introduced into a vial, in case of collecting the sample for further analysis (Figure 2, port 6, Table 2, Steps 10, 11) or to waste (in case of Cr(III) determination, during testing the system). The SP, HC, tubing and the EM were rinsed carefully with water between analyses (Table 1, Steps 12–18) and filled with air. To prevent drops of solution from remaining on walls, the whole system was washed daily with ethanol solution (50%, *v/v*) and again filled with air. 

Using the designed flow system, the evaporation process was carried out for the initial sample volume equal the syringe pump capacity (4 mL) and was continued in continuous way (without interrupting the preconcentration procedure) by introducing another (subsequent) portion of the sample to the syringe pump (Table 1, Steps 5, 6). In this way, the preconcentration process could be carried out continuously for different initial sample volumes. 

### 2.4. Preconcentration Factor 

Signal enhancement factor (EF) was proposed to be determined as a measure of the effectiveness of the preconcentration process. EF was determined based on the signal values measured before and after the evaporation procedure. To this aim, a substance (tracer) was proposed to be added to the sample solution. Signal (absorbance) A_0_ was measured by introducing the sample directly into the flow cell, then the evaporation process was started. After the evaporation process, a selected volume of the preconcentrated sample was taken from the module and signal A_pre_ was measured. EF was calculated as:

(1) $$\mathrm{EF}=\frac{A_{\mathrm{pre}}}{A_{0}}$$

EF was selected as reliable for determining the degree of the sample preconcentration due to the possibility of sample loss in the membraneless evaporation process. This factor can be applied for samples whose absorbance, measured at the selected wavelength, does not change after the sample preconcentration (i.e., the absorbance of the sample itself does not affect the absorbance value measured for the tracer). As a tracer, one can select a substance for which the signal does not coincide with the analyte signal. Since the accuracy of the results depends on the accuracy of the EF determination, it is important that signal A_0_ is stable and repeatable. 

### 2.5. Preliminary Studies 

**Figure 3:**
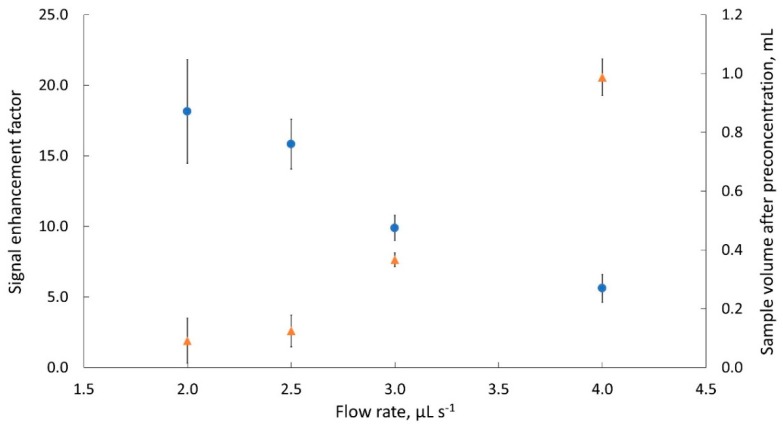
Dependences of the signal enhancement factor (●) and of the sample volume collected after the evaporation process (▲) (with SD intervals; *n* = 4) depending on the sample dispensing flow rate.

The preliminary tests included the selection of an appropriate flow rate for dispensing a sample into the EM. Cr(III) solution of concentration of 2.5 mmol L^−1^ was used as a sample because for this solution the analytical signal was stable and reproducible. The initial sample volume (V_s_) of 6 mL was subjected to the evaporation process. To make the preconcentration process as short as possible, the research was carried out at the thermostatic block heated up to 100 °C, under a vacuum of about 80 kPa. The tests were performed at four flow rates in the range from 2 to 4 μL s^−1^. For a given flow rate, the evaporation procedure was repeated four times. The determined mean EF values with the intervals of standard deviation (SD) are shown in Figure 3. At the same time, the volume of the sample remained after the evaporation process (V_pre_), was determined by taking the sample at a selected flow rate (10 µL s^−1^) from the EM, through the flow cell to the SP and measuring the duration of the sample signal. The obtained V_pre_ values together with the standard deviation intervals are also shown in Figure 3. 

Figure 3 shows that under selected conditions, as the flow rate increased, increased amount of the preconcentrated sample (from about 0.1 to 1.0 mL) remained in the vessel after evaporation and decreased EF values (from 18 to 5, respectively) were obtained. In addition, for smaller volumes, the EF values were characterized by worse precision. The best and acceptable precision (*n* = 4) was obtained at a flow rate of 3 μL s^−1^, namely SD = 0.9 and 0.024 mL for EF ≈ 10 and V_pre_ ≈ 0.4 mL, respectively. Thus, this flow rate value was decided to be used for further research. 

**Figure 4:**
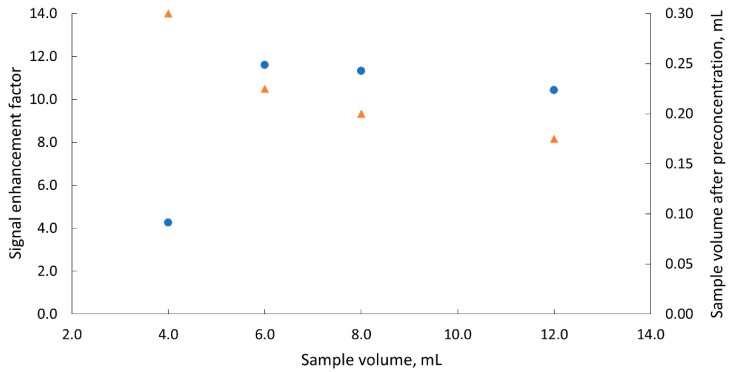
Dependences of the signal enhancement factor (●) and of the sample volume collected after the evaporation process (▲) on the initial sample volume subjected to evaporation; dispensing flow rate 3 μL s^−1^.

In the next step, it was decided to examine whether the V_s_ volume affects the EF factor and the volume V_pre_. To this end, 4 samples with a volume of 4 to 12 mL were evaporated under the same instrumental conditions. In this case evaporation was carried out once for the sample. The results are presented in Figure 4. It can be noticed that in the V_s_ range from 6 to 12 mL, the EF values ranged from 12 to 10, and the V_pre_ volume was about 0.20 mL. In further studies, volume V_s_ = 6 mL was taken for the evaporation process. Under these conditions, the evaporation process lasted about 34 min. 

**Figure 5:**
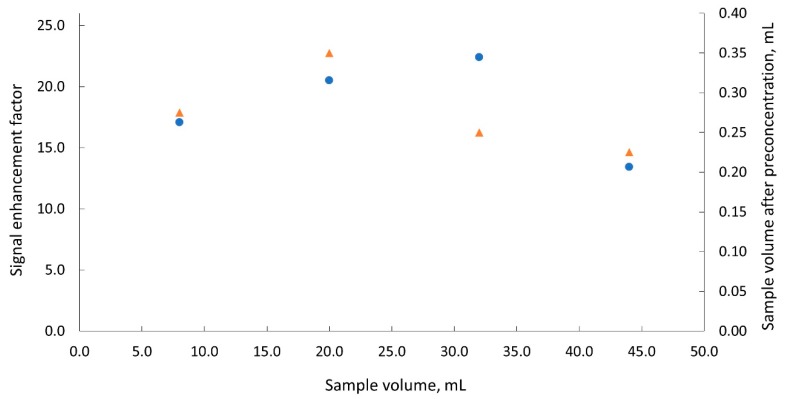
Dependences of the signal enhancement factor (●) and of the sample volume collected after the evaporation process (▲) on the initial sample volume subjected to evaporation; dispensing flow rate 4 μL s^−1^.

In order to check whether using the evaporation system, it is possible to achieve higher concentration coefficient values, tests were also carried out for a wider range of sample volume V_s_, from 8 to 44 mL, subjected to evaporation. To reduce the time needed to conduct the experiment, samples were dosed at a flow rate of 4 μL s^−1^. In this case, evaporation was carried out once for a sample. The results are shown in Figure 5. It can be stated that when V_s_ was increased more, even greater EF value, approaching 23, was possible to be achieved. However, in this case the preconcentration time increased significantly to several hours. 

### 2.6. Testing of the Flow-Based Preconcentration System 

The operation of the flow system with the EM module was tested on the example of Cr(III) determination in synthetic samples. To determine the concentration of analyte in the sample, C_S_, the calibration based on the standard addition method was applied. The standard with analyte of concentrations, ΔC_1_, …, ΔC_4_ was added to the sample in accordance with the standard addition method. All solutions were subjected to measurements separately after introducing them one after the other to the flow cell and the calibration graph was prepared. The analyte concentration in the first standard (ΔC_1_) was high enough to produce well repeatable signal A_0_. Then, the sample with the first standard addition (ΔC_1_) was introduced into the evaporation vessel, subjected to the evaporation process and transferred to the flow cell to measure the signal A_pre_. EF value was calculated from Equation (1) and the analyte concentration in the sample, C_s_, was determined using the equation:

(2) $$C_{s}=\frac{C_{\mathrm{pre}}}{\mathrm{EF}}-\Delta C_{1}$$

Samples with Cr(III) in concentrations between 0.1 and 0.5 mmol L^−1^ were subjected to the preconcentration process in different days. Each sample was evaporated three times in the same conditions. Evaporation was carried out at 100 °C, vacuum of 80 kPa and at a sample flow rate of 3 μL s^−1^ into the evaporation vessel. The signals were measured directly at 530 nm. Linear calibration graphs were obtained for Cr(III) in the concentration range 1.0–50.0 mmol L^−1^ (e.g., y = 0.0143x + 0.0006; R = 0.999; y—absorbance, x—concentration). The Table 2 presents the mean analytical results (with the values of coefficient of variance, CV and relative error, RE). 

Analyzing Table 2, it can be concluded that Cr(III) was determined with satisfactory accuracy: Relative error (RE) values exceeded 7% (11.8%) only in one case, for the lowest concentration of Cr(III). The results are also characterized by good precision, only in one case, CV value (also for the lowest Cr(III) concentration) exceeded 5% (8.4%), and intermediate precision (CV < 5.1%). With regard to accuracy, the highest RE values (5.5%–11.8%) were obtained for samples containing the lowest amounts of Cr(III). For samples containing more Cr(III), consistence of found and expected values was fully satisfactory. However, the method is dedicated to samples in which the analyte concentration is below the limit of quantification, therefore it can be assumed that for these concentration levels relative errors close to 10% may be acceptable.

### 2.7. Verification of the Flow Preconcentration System

The operation of the system was verified on the example of determination of zinc by capillary electrophoresis with spectrophotometric detection. Zn was determined in certified reference materials of drinking and waste water. To collect the entire sample volume after evaporation, the HC (Figure 2) was extended to 90 cm (i.d. 0.8 mm). First, the segment of air was introduced into the HC (to separate the sample from the solution in the pump and prevent the sample dilution, Table 1), then the sample (S_pre_) was introduced into the HC through the flow cell (to measure A_pre_) and finally (after changing the flow direction), the sample was directed to CE vial. To determine the EF value, Cr(III) at a concentration of 4.0 mmol L^−1^ was added to the sample. Limit of detection (LOD) and limit of quantification (LOQ) for the CE method were evaluated by calculating the signal-to-noise (S/N) ratio, considering the general rule that for LOD and LOQ the S/N ratio should be 3 and 10, respectively, which provided the concentration levels of 0.25 and 0.90 mg L^−1^, respectively. One should note that taking into account the EF delivered by the EM (EF = 10), the appropriate LOD and LOQ levels for Zn determination were 0.025 and 0.09 mg L^−1^, respectively. 

The mean values of the determined Zn concentration and the corresponding values of the confidence interval (*n* = 3, α = 0.05) were calculated for drinking water (2.4 ± 0.5) mg L^−1^ and wastewater (0.80 ± 0.15) mg L^−1^. To evaluate the agreement between obtained and certified concentrations, the values were compared with each other using the Student’s t-test (α = 0.05). Certified values were treated as true values. It was confirmed that the determined concentrations were consistent with the certified values (2.49 ± 0.06) mg L^−1^ and (0.862 ± 0.010) mg L^−1^, respectively. Generally, in the certified reference material samples Zn was determined with precision better than 6% (CV). The results showed the possibility of using the developed approach (for sample preconcentration and determination of the preconcentration degree) for the analyte determination using another method. The total analysis time included time necessary for the sample preconcentration and time of CE analysis. In the case of Zn determination, it was about 40 and 15 min, respectively.

To sum up, it can be stated that the developed mechanized flow system with the module for evaporation has a chance to be used to preconcentrate samples with the ability to determine the degree of sample preconcentration. The preconcentrated sample can be introduced into another detection system to determine the concentration of the specific analyte/analytes. For this purpose, e.g., capillary electrophoresis technique can be used, which allows the separation of analytes, while at the same time very small sample volumes can be used for analysis. This technique with spectrophotometric detection is characterized by relatively high values of the detection limit. Therefore, the use of the developed flow system with the preconcentration module creates greater possibilities for determining various analytes. Compared to other sample preconcentration systems based on the membraneless evaporation, its advantage may be the ability to easily adapt to preconcentrate different samples of various volumes. In the developed system, preconcentration time depends on the initial sample volume, the flow rate of sample introduced to the EM and the applied conditions of pressure and temperature. These conditions should be selected to a sample individually. The use of a tracer makes it possible to determine the signal enhancement factor for a particular determination; however, attention should be paid to the selection of the tracer and its concentration, so that it is possible to determine the analyte with acceptable accuracy. Further research can include the possibility of combining the developed flow-based evaporation system with CE instrument and selecting the CE analysis conditions to determine more analytes.

Compared to the membraneless evaporation system described in the literature [30], using the developed approach better precision of the results was obtained (8.4%, CV) but the evaporation time was longer. For the reported system [30] the values of enrichment factors were not given. Regarding other literature examples of the membraneless evaporation [31,32,33], they were characterized by better precision and shorter time of analysis; however, they were designed to determine (volatile) analyte in the evaporated fraction. 

## 3. Materials and Methods 

### 3.1. Instruments and Materials

The flow system consisted of bidirectional syringe pump SIChrom (FIAlab, Seattle, WA, USA) with 10-position selection valve (VICI Valco Instruments, Houston, USA), light source (deuterium and halogen lamps) DH-2000 (Ocean Optics, Dunedin, FL, USA), light fibers, spectrometer USB 4000 (Ocean Optics, USA), Ultem flow cell SMA-Z—10 mm optical path, and PTFE tubing, i.d. 0.8 or 0.5 mm (IDEX Health & Science, Rohnert Park, California, CA, USA). FIAlab software (FIAlab, Seattle, WA, USA) served in data acquisition, signals visualization and measuring. The preconcentration module included aluminum heating block (KPS Elektronika Laboratoryjna, Olsztyn, Poland), vacuum pump ME 2C NT + 2AK (Vacuubrand, Wertheim, Germany), temperature controller Transmit PID G6 (Termipol, Lubliniec, Poland), prsssure gauge ZSE4/ISE4 (SMC, Chiyoda, Japan), and electronic controller 60/16 (KPS Elektronika Laboratoryjna, Olsztyn, Poland). 

The capillary electrophoresis analyses were performed using the PA/800 Plus system (Beckman-Coulter, Miami, Florida, FL, USA), equipped with the UV detector operating at 200 nm in an indirect detection mode. Separations were carried out in the bare fused silica capillary (75 µm i.d., total length of 60.2 cm, effective length of 50.2 cm), thermostated to 30 °C. Every working day, before the first analysis the capillary was rinsed (20 psi = 137.9 kPa) in a sequence with MeOH (20 min), 1 mol L^−1^ HCl (5 min), water (1 min), 1 mol L^−1^ NaOH (5 min), 0.1 mol L^−1^ NaOH (20 min), water (2 min), and background electrolyte (2 min). Between runs, the capillary was flushed only with BGE for 3 min (20 psi, 137.9 kPa). Samples and calibration solutions were stored in the thermostated sample garage (25 °C) and they were hydrodynamically injected (0.5 psi = 3.45 kPa, 5 s) into the capillary before applying the separation voltage of 20 kV (anode at inlet; 0.5 min ramping time) for 10 min. 

### 3.2. Reagents and Solutions 

Standard solutions and synthetic samples containing Cr(III) were prepared using chromium(III) nitrate 9-hydrate, Cr(NO_3_)_3_ · 9H_2_O, (POCh SA, Gliwice, Poland). The Cr(III) stock solution (0.05 g L^−1^) was prepared by dissolving appropriate amount of the Cr(NO_3_)_3_ · 9H_2_O in water. Standard solutions and synthetic samples were prepared by appropriately diluting the stock solution with water. Certified reference materials of drinking water (EnviroMAT Drinking water, high (EP-H) and wastewater (EnviroMAT Wastewater, high (EU-H) (SCP SCIENCE, Quebec, ON, Canada) were used for the system verification. The materials were diluted with water according to the producer instructions. Zn calibration solutions were prepared by appropriate dilution of Zn standard solution (1000 mg, Merck, Darmstadt, Germany) with water. Reagents of analytical grade were used. Deionized water (0.05 µS cm^−1^) obtained from HLP5sp system (Hydrolab, Straszyn, Poland) was used throughout the study. For the determination using capillary electrophoresis method, the background electrolyte (BGE) composition was optimized for the purpose and it consisted of 15 mmol L^−1^ imidazole (Bioshop, Burlington, Canada), 2 mmol L^−1^ 18-crown-6 ether (Sigma Aldrich, Saint Louis, MO, USA), 5% (*v*/*v*) methanol (Sigma Aldrich, USA), 5% (*v*/*v*) acetonitrile (Sigma Aldrich, Saint Louis, MO, USA), and it was adjusted to pH = 3.7 with acetic acid (Sigma Aldrich, Saint Louis, MO, USA). BGE was then filtered through the regenerated cellulose syringe filter (0.45 µm) and—before analyses—degassed by centrifugation. Fifty microliters of each of the preconcentrated sample was diluted with 40 µL of BGE and 10 µL of deionized water. The Zn calibration solutions were prepared the same way to keep the BGE dilution constant. The calibration plot was constructed by taking the time-corrected peak area as the analytical signal. 

## 4. Conclusions 

In summary, a mechanized flow system with an original module was developed in which the sample preconcentration based on a membraneless evaporation takes place in a continuous mode. Using the system and the proposed mode of determining the signal enhancement factor, samples of various initial volumes can be preconcentrated and the degree of sample preconcentration can be determined. Using different evaporation conditions, various values of the signal enhancement factor (from several to 20) were obtained. In the optimized conditions for Cr(III) determination, it was possible to obtain signal enhancement factors of around 10 with a precision (CV) of less than 10%. Preconcentrated samples can be taken for further analyses. The operation of the flow system with the preconcentration module was positively verified on the example of the determination of Zn in certified reference materials of drinking water and wastewater using capillary electrophoresis method. 

## Figures and Tables

**Table 1:** Procedure developed for the preconcentration of 6 mL of sample in the proposed flow system; S_pre_—preconcentrated sample signal, SV—selection valve, SP—syringe pump, HC—holding coil, EM—evaporation module, T—tubing, CE—capillary electrophoresis method; *—for Zn determination, **—for Cr(III) determination.

Step	Vacuum Pump	SV Position	SP Flow Rate, μL s^−1^	Substance	Volume, μL	Action
1	Off	4	200	Sample	1000	Introducing the sample into the SP (washing T3, HC and SP with sample)
2	Off	7	200	Sample	1000	Transport of the sample to waste
Two repetitions of stages 1–2						
**Preconcentration**						
Turning on the vacuum pump						
3	On	4	200	Sample	4000	Aspiration of the sample to the SP
4	On	1	3	Sample	4000	Introducing the sample into the EM Sample signal measurement
5	On	4	200	Sample	2000	Aspiration of the sample to the SP
6	On	1	3	Sample	2000	Introducing the sample into the EM; Sample signal measurement
Turning off the vacuum pump						
7	Off	1	0	-	-	Delay 30 s
**Signal S_pre_ Measurement**						
8	Off	10	200	Air	1000	Aspiration of air to the HC
9	Off	2	10	S_pre_	500	Aspiration of the S_pre_ to the flow cell; S_pre_ signal measurement
**Collecting the sample, Washing SP, HC, Tubing and EM**						
10	Off	6	50	S_pre_	600 */0 **	Transport of the S_pre_ to a vial for further analysis
11	Off	7	200	Solutions and Air	900 */1500 **	Transport of the solutions and air to waste
12	Off	5	200	Carrier (H_2_O)	1000	Introducing water into the SP
13	Off	7	200	Carrier (H_2_O)	1000	Transport of water to waste
Two repetitions of stages 12–13						
14	Off	5	200	Carrier (H_2_O)	4000	Introducing water into the SP
15	On	1	200	Carrier (H_2_O)	4000	Introducing water into the EM
16	On	1	0	-	-	Delay 30 s
17	Off	2	200	Carrier (H_2_O)	4000	Introducing water into the SP
18	Off	7	200	Carrier (H_2_O)	4000	Transport of water to waste
Two repetitions of stages 14–18						
Repetition of stages 14-18 using air instead of water (SV position 10, to remove water from T1, EM, and T2)						

**Table 2:** Results of the determination of Cr(III) using the developed flow system (*n* = 3).

No.	Cr(III) Concentration, mmol L^−1^		│RE│, %	CV, %
	Expected	Found		
1	0.10	0.11	6.9	2.0
2		0.11	11.8	2.1
3		0.11	5.5	8.4
4	0.20	0.21	3.4	1.0
5		0.20	0.6	1.6
6		0.21	3.3	2.9
7	0.30	0.30	1.6	1.3
8		0.29	3.8	1.4
9		0.30	0.2	2.5
10	0.40	0.38	4.1	1.4
11		0.38	5.6	1.3
12		0.40	0.8	4.9
13	0.50	0.47	5.2	1.4
14		0.52	4.4	1.2
15		0.49	1.2	4.6
